# Adequacy of recommendations for adverse event management in national and international treatment guidelines for rifampicin-susceptible tuberculosis: a systematic review

**DOI:** 10.1016/j.eclinm.2025.103148

**Published:** 2025-03-18

**Authors:** William Burman, Jayne Ellis, Gila Hale, Katherine Hill

**Affiliations:** a Public Health Institute at Denver Health, Denver, CO, USA; b Department of Medicine, University of Colorado Anschutz Medical Campus, Aurora, CO, USA; c Clinical Research Department, London School of Hygiene and Tropical Medicine, London, United Kingdom; d Infectious Diseases Institute, College of Health Sciences, Makerere University, Kampala, Uganda; e University of St. Andrews, North Haugh, St. Andrews, United Kingdom

**Keywords:** Tuberculosis treatment, Adverse events, Treatment guideline

## Abstract

**Background:**

Adverse events during tuberculosis treatment are common and are a major challenge for patients and front-line care providers. We did a systematic review of treatment guidelines for rifampicin-susceptible tuberculosis to evaluate the adequacy of recommendations for adverse event management.

**Methods:**

We searched websites, guideline registries, PubMed, and mobile health Apps to identify treatment guidelines published from October 2004 to October 2024. We recorded the presence and evidence base for specific recommendations for management of nausea/vomiting, hepatotoxicity, skin reactions, neuropathy, visual changes, drug fever, and arthralgias.

**Findings:**

We included 47 guidelines: 25 from high-burden countries, 12 international and prominent national guidelines, and 10 non-governmental guidelines. 37 guidelines (79%) included recommendations for managing adverse events: 24 (96%) of guidelines from high-burden countries, eight (80%) of those from non-governmental organizations, and four (33%) of international and prominent national guidelines. Four recommendations had formal ratings of supporting evidence.

**Interpretation:**

International and prominent national guidelines frequently lack recommendations for adverse event management or had non-specific recommendations. Research on prevention and management of common and serious adverse events should be a priority for improving the patient’s experience and the outcomes of tuberculosis treatment.

**Funding:**

This research was supported by 10.13039/100010269Wellcome Trust Clinical Grants.

**Research in context**

**Evidence before this study**

Adverse events are common during tuberculosis treatment, but there have not been prior systematic reviews of the adequacy of recommendations for management of adverse events in tuberculosis treatment guidelines.

**Added value of this study**

Recommendations for management of adverse events were commonly present in guidelines from high-burden countries, but often absent or non-specific in prominent international and national governmental guidelines. None of the recommendations provided were based on strong evidence. Guidelines frequently lacked features that would promote ease of use: clear labeling, functional weblinks for additional information, tables or algorithms.

**Implications of all the available evidence**

Treatment guideline should provide recommendations on management of adverse events, even if the evidence for doing so is of low quality. Research on prevention and management of adverse events should be a priority for improving the patient’s experience and the outcomes of tuberculosis treatment. Greater attention to ease of use of recommendations for adverse event management would help busy clinical staff.

## Introduction

With over 10 million cases and 1 million deaths in 2023, rifampicin-susceptible tuberculosis remains a major public health problem.^1^ Treatment-related adverse events are common^2^, ^3^, ^4^, ^5^, ^6^, ^7^ and problematic during the treatment of rifampicin-susceptible TB using the standard regimen (isoniazid, rifampicin, pyrazinamide, and ethambutol). Adverse events are associated with missed doses and treatment interruptions,^8^, ^9^, ^10^ regimen changes and treatment extensions,^11^, ^12^, ^13^ treatment non-completion,^14^ and increases in treatment failure, recurrence, and death.^2^^,^^5^^,^^15^ Moreover, some populations – elderly persons and persons with HIV co-infection,^16^ diabetes^17^ and alcohol use disorder^18^ – are at higher risk for drug intolerance and adverse events, translating to worse outcomes in programmatic settings.^11^^,^^12^

Common treatment-related adverse events are nausea/vomiting, hepatotoxicity, skin rash, peripheral neuropathy, visual disturbances, drug fever, and arthralgias.^3^ While treatment-related adverse events are reported in clinical trials, there has been a paucity of trials devoted to the prevention or management of adverse events.^19^ As a result, patients and front-line care providers may lack timely and easily accessible recommendations about how to manage common adverse events during the treatment of rifampicin-susceptible tuberculosis. Therefore, we did a systematic review of recommendations for adverse event management in national and international TB treatment guidelines. Identifying gaps in treatment guidelines may be useful in improving subsequent versions of those guidelines and in setting research priorities.

## Methods

This review followed the Preferred Reporting Items for Systematic Review and Meta-Analysis (PRISMA) guidelines.^20^ We had a confirmed receipt of the protocol at the PROSPERO site on 8 September 2024 but have not received a registration number. We searched PubMed on 1 October 2024 for papers including key terms of “tuberculosis” AND “guideline OR guidelines” (see Supplementary Materials, page 6) from October 2004 to October 2024. We searched on-line treatment guideline repositories in October 2024 (see Supplementary Materials, page 7). One of our objectives was to evaluate treatment guidelines from countries with high numbers of tuberculosis cases. We used the World Health Organization’s list of 30 high TB burden countries from 2021^21^ and sought to obtain the most recent treatment guidelines for treatment of rifampicin-susceptible tuberculosis from these countries through internet searches and the authors’ contacts in national and international tuberculosis programs. Finally, we searched in three ways for mobile health Apps that might contain guidelines on adverse event management: i) the Apple and Google App stores (for “tuberculosis OR TB OR *Mycobacterium tuberculosis* AND Treatment OR guidelines OR side effects OR adverse events”), ii) Chat GPT, and iii) a previous publication on Apps for tuberculosis.^22^ Because availability of mobile health Apps may differ by country, we repeated searches of the Apple and Google App stores from the United States, the United Kingdom, and Uganda.

### Search strategy and selection – inclusion/exclusion criteria

We included treatment guidelines for the management of rifampicin-susceptible active tuberculosis. We did not include treatment guidelines for rifampicin-resistant tuberculosis, treatment to prevent tuberculosis, or city, state, or provincial treatment guidelines. We categorized treatment guidelines into those from high-burden countries, those from international organizations or countries whose guidelines are often cited in other national guidelines and hence, may be used as key reference documents by health workers globally (“international and prominent national”), and those from non-governmental organizations. We used Chat GPT to translate guidelines that were not available in English or Spanish, and we repeated the translation using Perplexity (a second artificial intelligence tool, www.perplexity.ai) to assess the translations done by Chat GPT. To assess for changes over time in international and prominent national treatment guidelines, we evaluated the last two versions of guidelines from the World Health Organization (WHO), the European Union, and the United States. For other guidelines, if there were multiple versions over the past 20 years, we used the most recent version.

Because treatment for active tuberculosis always involves multiple agents, we defined recommendations for adverse event management as those that provided guidance on the management of specific clinical syndromes (nausea/vomiting, hepatotoxicity, skin reactions, neuropathy, visual changes, drug fever, and arthralgia). Therefore, a listing of possible side effects of individual drugs did not meet our definition of recommendations for adverse event management. We focused data extraction on two areas: i) the presence and evidence base of recommendations for managing treatment-related adverse events, and ii) the ease of use of the recommendations. We recorded the use of a specific framework for developing treatment guidelines, such as the Grading of Recommendations Assessment, Development and Evaluation (GRADE) framework,^23^ and the rating of the evidence for a recommendation, when available.

To assess ease of use, we used the criteria of the Appraisal of Guidelines, Research and Evaluation, (AGREE) II instrument^24^^,^^25^ though we did not assign scores to the AGREE II items related to ease of use (“Specific and unambiguous recommendations”, “Key recommendations easily identifiable”). Instead of numeric scores we recorded whether the guideline had a table of contents or a webpage listing that made it clear where recommendations for managing adverse events could be found, whether the guideline had tables and/or figures, and whether web-links in the documents or webpages were functional. To assess specificity of the recommendations we recorded whether specific medications and doses were provided for treating selected adverse events (e.g., nausea/vomiting, neuropathy) and whether drug–drug interactions that might impact treatment of an adverse event were identified.

One reviewer (WB) reviewed the titles from the PubMed search, selected articles for full-text review, and selected those to be included in the systematic review. One reviewer (WB, JE, GH, or KH) reviewed the treatment guidelines included in the review using a standard data abstraction tool. All authors looked for and assessed the Apps identified through the search. To verify the quality and consistency of data extraction, four guidelines (9%) were selected to be reviewed by another author (JE). Two authors (JE, GH) compared translations done by Chat GPT to those done by Perplexity.

We used Microsoft Excel for data collection. We performed a descriptive analysis of categorical variables, such as inclusion of recommendations for adverse events. This research was supported by a Wellcome Trust Clinical PhD Fellowships (Grant 203905/Z/16/Z to JE and Grant 223499/Z/21/Z to KH).

### Ethics

This protocol was not submitted for ethical review as it is a systematic review of treatment guidelines, not patients or personal health information.

### Role of funding source

The Wellcome Trust did not have any role in study design, data collection, data analyses, interpretation, or writing of the report.

## Results

**Fig. 1:**
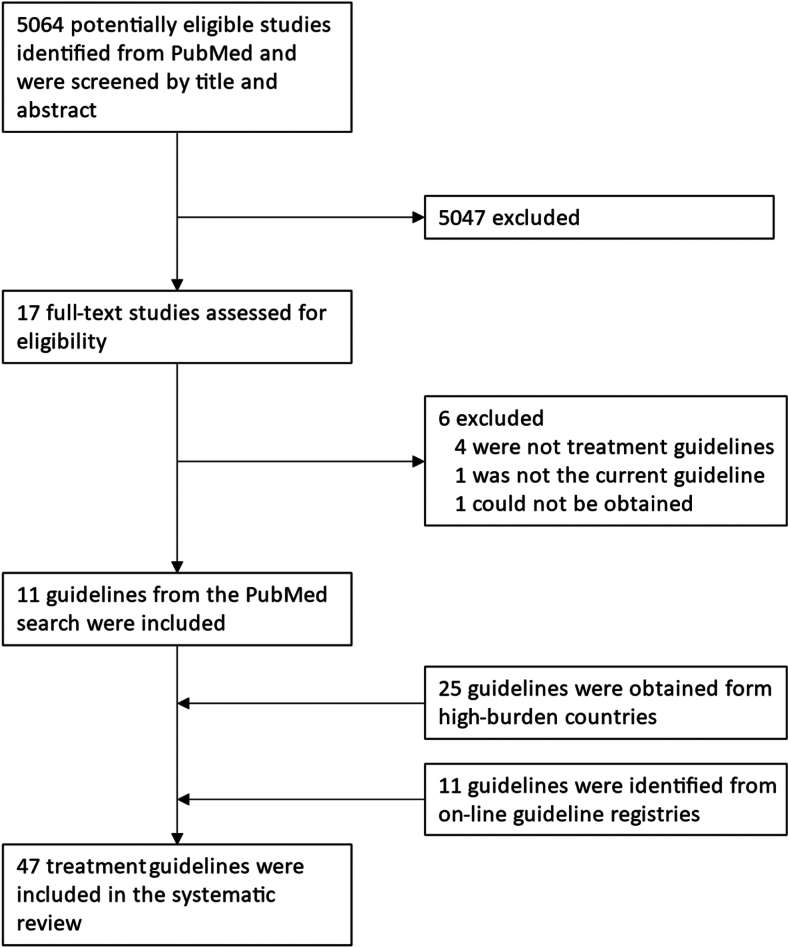
**Study selection.** PRISMA flow diagram of study selection.

Of 5264 titles identified in the search, 17 underwent full-text review, of which 11 met inclusion criteria (Supplementary Materials, page 6). Most of the guidelines included in the systematic review were identified from on-line treatment guideline repositories or by internet searches for national guidelines (Fig. 1). In total, we included 47 treatment guidelines in our systematic review: 25 guidelines from high-burden countries for tuberculosis (eight of which were translated by ChatGPT), 12 international or prominent national guidelines, and 10 guidelines from non-governmental organizations (Table 1). We were unable to obtain guidelines from 5 of the 30 high-burden countries (17%). Translations by the two artificial intelligence tools (Chat GPT and Perplexity) were very similar, though we noted that Chat GPT provided more detailed information regarding adverse event management in three of the guidelines (Democratic Republic of the Congo, Republic of Korea, and Thailand), in part due to more complete translation of material within tables.

**Table 1:** Treatment guidelines included in the systematic review, by type.

Organization (reference)	Name of the guideline	Year	Recommendations for management of adverse events
International and prominent national guidelines			
Stop TB partnership childhood TB subgroup^26^	Chapter 2: Anti-tuberculosis treatment in children	2006	Present
Tuberculosis coalition for technical assistance^27^	International standards for tuberculosis Care	2006	Not present
United States of America^28^	ATS/CDC/IDSA Guidelines for Treatment of Drug-Susceptible Tuberculosis	2016	Present
European Union^29^	European Union Standards for Tuberculosis Care 2017 update	2017	Not present
World Health Organization^30^	WHO treatment guidelines for isoniazid resistant tuberculosis	2018	Not present
World Health Organization^31^	WHO consolidated guidelines on tuberculosis: module 4: treatment: drug-susceptible tuberculosis treatment	2022	Not present
World Health Organization^32^	WHO consolidated guidelines on tuberculosis: module 5: management of tuberculosis in children and adolescents	2022	Not present
Canada^33^	Canadian Tuberculosis Standards - Canadian Thoracic Society	2022	Not present
Australia^34^	Tuberculosis CDNA National Guidelines for Public Health Unit	2022	Not present
Spain^35^	Consensus document regarding antiretroviral treatment among adults	2023	Not present
United States Department of Health and Human Services^36^	Guidelines for the Prevention and Treatment of Opportunistic Infections in Adults and Adolescents With HIV	2024	Present
United Kingdom^37^	National Institute for Health and Excellence: Tuberculosis	2024	Present
Non-governmental guidelines			
British Infection Society^38^	British Infection Society guidelines for the diagnosis and treatment of tuberculosis of the central nervous system in adults and children	2009	Present
British Thoracic Society^39^	Guidelines for the prevention and management of *Mycobacterium tuberculosis* infection and disease in adults patients with chronic kidney disease	2010	Not present
Sociedad Espanola de Neumologıa y Cirugía Toracica^40^	Consensus document regarding diagnosis, treatment, and prevention of tuberculosis	2010	Present
International Union Against TB and Lung Disease^41^	Management of Tuberculosis: A Guide to Essential Practice	2019	Present
European AIDS Clinical Society^42^	Guidelines Version 10.0	2022	Not present
British HIV Association^43^	British HIV Association guidelines for the management of tuberculosis in adults living with HIV	2023	Present
European Society of Clinical Microbiology and Infectious Diseases^44^	Clinical standards for the management of adverse effects during treatment for TB	2023	Present
Up to date^45^	Treatment of drug-susceptible tuberculosis in non-pregnant adults without HIV infection	2024	Present
South Africa HIV clinicians society^46^	Management of drug-induced liver injury in people with HIV treated for tuberculosis: 2024 update	2024	Present
Medecins Sans Frontiere (MSF)/Partners in Health^47^	Tuberculosis: Practical guide for clinicians, nurses, laboratory technicians and medical auxiliaries	2024	Present
Guidelines from high-burden countries			
Bangladesh^48^	National guideline and operational manual for tuberculosis	2021	Present
Brazil^49^	Brazilian Thoracic Association Guidelines on Tuberculosis	2009	Present
China^50^^a^	China Tuberculosis Prevention and Control Technical Specifications	2020	Present
Congo (Democratic Republic of Congo)^51^^a^	Management of tuberculosis	2022	Present
Ethiopia^52^	Guidelines for management of TB, DR-TB and leprosy in Ethiopia, 6th edition	2017	Present
India^53^	INDEX-TB Guidelines. Guidelines on extra-pulmonary tuberculosis for India.	2016	Not present
Indonesia^54^^a^	Indonesian Health Ministerial Decree No.67	2016	Present
Kenya^55^	Integrated guideline for tuberculosis, leprosy and lung disease	2021	Present
Korea (Republic of Korea)^56^^a^	Korean Guidelines for Tuberculosis	2024	Present
Liberia^57^	National Tuberculosis Management Guidelines, Liberia	2019	Present
Lesotho^58^	National Guidelines for Drug Susceptible Tuberculosis	2019	Present
Mongolia^59^^a^	Mongolia TB treatment guidelines	2009	Not present
Mozambique^60^^a^	Mozambique National TB Protocols	2019	Present
Myanmar^61^	Guideline for drug sensitive TB management in Myanmar	2020	Present
Namibia^62^	National Guidelines for the Management of Tuberculosis Fourth Edition	2019	Present
Nigeria^63^	National tuberculosis, leprosy and Buruli ulcer management and control guidelines	2023	Present
Pakistan^64^	National guidelines for the control of tuberculosis in Pakistan	2019	Present
Philippines^65^	TB (DS & DR) and Latent TB Screening, Diagnosis and Management Pocket Guide	2024	Present
Sierra Leone^66^	National guidelines for clinical and programmatic management of tuberculosis in Sierra Leone	2024	Present
South Africa^67^	National guidelines on the treatment of tuberculosis infection	2023	Present
Tanzania^68^	Manual for management of tuberculosis and leprosy in Tanzania	2020	Present
Thailand^69^^a^	Thailand National Tuberculosis Control Program Guideline	2018	Present
Uganda^70^	Uganda National Tuberculosis and Leprosy Control Programme Manual for Management and Control of Tuberculosis and Leprosy. 3rd Edition	2017	Present
Vietnam^71^^a^	Guidelines for Diagnosis, Treatment, and Prevention of Tuberculosis	2024	Present
Zambia^72^	National tuberculosis and leprosy programme	2022	Present

a Guidelines from China, Congo, Indonesia, Korea, Mongolia, Mozambique, Thailand, and Vietnam were translated using ChatGPT.

Our search identified 21 Apps related to tuberculosis (see Supplementary Materials, page 10). Of these, three Apps had recommendations for treatment-related events: two (IDSA [Infectious Diseases Society of America] Practice Guidelines, MSF [Médecins Sans Frontières] Clinical Guidelines) had links to the on-line version of tuberculosis treatment guidelines that had already been included in our review^28^^,^^47^; the third (the Georgia TB Reference Guide)^73^ had recommendations for baseline clinical and laboratory tests to monitor for adverse events but did not have recommendations for adverse event management. In summary, the search for mobile health Apps did not identify any treatment guidelines that were not available from internet searches or PubMed.

Of the 47 guidelines, 37 (79%) included any recommendations for managing treatment-related adverse events (Table 2). Nearly all treatment guidelines from high-burden countries (23, 92%) and those from non-governmental organizations (8, 80%) included such recommendations, compared to 4 (33%) of the international and prominent national guidelines (See Supplementary Materials, pages 11–13). Notably, none of the current guidelines from the WHO,^74^ the European Union,^29^ Canada,^33^ or Australia^34^ included any recommendations for managing adverse events. Furthermore, the United Kingdom’s National Institute for Health and Excellence guidelines only included recommendations for one adverse event, hepatotoxicity.^37^

**Table 2:** Inclusion of recommendations for managing treatment-related adverse events, overall and by type of event (n, %).

	Total (n = 47)	International and prominent national guidelines (n = 12)	Non-governmental guidelines (n = 10)	High-burden country guidelines (N = 25)
Recommendations managing adverse events present	36 (77)	4 (33)	8 (80)	23 (92)
Risk factors for adverse events identified	16 (34)	0	2 (20)	14 (56)
Recommendations for specific adverse events^a^				
Nausea/vomiting	25 (53)	1 (8)	5 (50)	19 (76)
Non-pharmacological measures	25 (53)	1 (8)	5 (50)	19 (76)
Medical therapy	15 (32)	1 (8)	4 (40)	10 (40)
Hepatotoxicity	35 (74)	4 (33)	8 (80)	23 (92)
Skin reactions	26 (55)	2 (17)	5 (50)	19 (76)
Neuropathy	27 (57)	1 (8)	5 (50)	21 (84)
Visual changes	27 (57)	1 (8)	4 (40)	22 (88)
Drug fever	14 (30)	1 (8)	2 (20)	11 (44)
Arthralgias	21 (45)	0	2 (20)	19 (76)

a See Supplementary Materials, pages 11–13 and 14–15 for details.

16 guidelines (34%) included a review of risk factors for adverse events, most often those from high-burden countries (Table 2, Supplementary Materials, pages 14–15). The pattern for the inclusion of recommendations for specific adverse events was similar, being most often present in guidelines from high-burden countries and non-governmental organizations (Table 2).

Use of a formal framework for rating of the strength and evidence for recommendations was more common among international and prominent national guidelines (7/12, 58%) and non-governmental guidelines (3/9, 33%) than among those from high-burden countries (1/25, 4%).^53^ Of the recommendations for managing adverse events, four – all dealing with management of suspected drug-induced liver injury among persons with HIV-related tuberculosis – had formal ratings of the strength of the recommendation and supporting evidence (three strong recommendations and one moderate recommendation based on “moderate-quality evidence” (one recommendation) and “expert opinion” (three recommendations).^36^^,^^43^ Nearly all the other recommendations did not have a formal rating of the strength of supporting evidence. 13 guidelines (28%) included recommendations for research to improve the evidence base for tuberculosis treatment; only 2 of these called for research on adverse event management.^37^^,^^44^

We evaluated the changes over the past 20 years in recommendations for adverse event management in three prominent guidelines (WHO, European Union, and United States). Adverse event management was included in the 2010 WHO guidelines^75^ and then removed completely in 2022,^31^ was not included in either of the most recent European Union standards for tuberculosis care,^29^^,^^76^ and there were only minor changes in recommendations in the United States guidelines between 2003^77^ and 2016^28^ (see Supplementary Materials, pages 16–17). During the review process of this systematic review, an updated version of the U.S. tuberculosis treatment guideline was published.^78^ We did not amend our study protocol and did not replace the data elements from the 2016 guideline in our tables and analyses with those from the 2025 update. However, given the prominence of this guideline, we reviewed the 2025 update and noted that it neither includes any recommendations for adverse event management nor calls for research on this gap in the evidence base for treatment guidelines.

Given their frequency and adverse impacts on the patient’s experience of treatment and its outcomes, we evaluated recommendations on the management of nausea/vomiting and hepatotoxicity (drug induced liver injury) in more detail.^3^^,^^6^ Half of the guidelines included recommendations on the management of nausea/vomiting (25, 53%). Non-pharmacologic measures were often recommended, most commonly dosing with food or dosing at bedtime (See Supplementary Materials, pages 18–19). 15 guidelines (33%) included recommendations for classes of medications for gastrointestinal symptoms (anti-emetic medications, antacids, and proton-pump inhibitors), but only six guidelines (13%) recommended specific medications, of which three (7%) recommended specific doses (one guideline provided dosing recommendations for children).^47^

35 guidelines (76%) included recommendations for managing suspected drug-induced liver injury, more often among guidelines from high-burden countries (23, 92%) and non-governmental organizations (8, 80%) than among international and prominent national guidelines (4, 33%) (Table 2). 28 guidelines (61%) included specific criteria for stopping tuberculosis treatment, most often using laboratory criteria (e.g., hepatic transaminase levels > 3 × upper limit of normal [ULN] in the presence of symptoms or >5 × ULN in the absence of symptoms). 29 guidelines (63%) recommended re-challenge once the clinical and laboratory abnormalities had improved: 25 guidelines recommended sequential re-challenge with full doses of individual medications, three recommended sequential re-challenge with dose-escalation of individual medications),^38^^,^^40^^,^^43^ and one recommended re-challenge with full doses of all medications at once.^47^ Only 5 guidelines recommended routine re-challenge with pyrazinamide, if rifampicin and isoniazid re-challenge had been well-tolerated; others suggested limiting pyrazinamide re-challenge to patients without severe drug-induced liver disease or in patients with severe forms of tuberculosis (Supplementary Materials, pages 20–21).

The use of tables, figures, and algorithms can help users find key recommendations quickly and work through complex clinical decision-making, as in the management of suspected drug-induced liver injury. Half of the guidelines contained at least one table or figure devoted to adverse event management (Table 3, Supplementary Materials, pages 14–15).

**Table 3:** Ease of use and specificity of recommendations among guidelines providing recommendations for adverse event management (n, %).

	Total (n = 35)	International and prominent national guidelines (n = 4)	Non-governmental guidelines (n = 8)	High-burden country guidelines (N = 23)
Guidelines containing at least one table and/or figure regarding adverse events, n (%)	23 (66)	2 (50)	8 (100)	13 (52)
Number of tables and/or figures, average (range)	2.6 (1–9)	1 (1)	3.0 (1–8)	2.7 (1–9)
Table of contents providing clear directions to recommendations for adverse events, n (%)	25 of 29 assessable^a^ (86)	3	8	14^a^
Presence of functional web-links to additional information on adverse events, n (%)	5 of 29 assessable (17)^a^	2	3	0^a^
Specific medications recommended	27 (21 only for pyridoxine)	0	3 (1 only for pyridoxine)	22 (18 only for pyridoxine)
Specific doses recommended	18 (14 only for pyridoxine)	NA	2 (1 only for pyridoxine)	16 (13 only for pyridoxine)
Drug–drug interactions in managing adverse events identified	3	0	1^b^	2^c^

See Supplementary Materials, pages 14–15 for details.

a Could not be assessed for six guidelines that were translated by ChatGPT and had recommendations for managing adverse events.

b Decreased absorption of fluoroquinolone antibiotics when taken with medications containing divalent cations (e.g., antacids).^28^

c Caution about use of ondansetron with other medications that can prolong QTc.^60^^,^^64^

The inclusion and numbers of such tables and figures paralleled the presence of recommendations for adverse event management, being more common in non-governmental and high-burden country guidelines. There were gaps in providing clear direction to sections on adverse event management. For example, the United States treatment guideline included adverse event management in a section entitled “Practical aspects of treatment”.^28^ Only 5 guidelines included functional weblinks to additional information. 27 guidelines (59%) contained recommendations for specific medications for treatment of adverse events, 19 of which included recommendations for specific doses. Notably, most of these specific recommendations were for pyridoxine for the treatment of neuropathy (Table 3, Supplementary Materials pages 14–15). Three guidelines included notes about drug–drug interactions that may affect treatment of adverse events,^28^^,^^60^^,^^64^ though no guidelines noted the drug–drug interactions between rifampicin and commonly used anti-emetic medications (ondansetron, metoclopramide)^79^^,^^80^ and a commonly used proton-pump inhibitor (omeprazole).^81^

## Discussion

Recommendations for management of adverse events were present in most (79%) guidelines for treatment of rifampicin-susceptible tuberculosis included in our systematic review. Guidelines from high-burden countries and non-governmental organizations often included recommendations for adverse event management; however, it is concerning that only 4 (33%) of the international and prominent national guidelines, which are frequently used by health workers globally, had any such recommendations. The lack of management recommendations for nausea in international and prominent national guidelines (only 25% had any recommendations for managing nausea) is particularly striking in that this may be the most common treatment-related adverse event.^82^^,^^83^ The gaps and heterogeneity of recommendations in our review demonstrates the need for much greater attention to adverse event management in treatment guidelines, given the frequency and impact of these events on the patient experience of treatment, and treatment outcomes. Finally, the presentation of recommendations for adverse event management could also be improved with greater use of tables and algorithms, appropriate weblinks, and more specific treatment recommendations.

Two prior systematic reviews of tuberculosis treatment guidelines have been published, though neither included any comments about the inclusion of recommendations for adverse event management. One review focused on diagnosis and treatment of tuberculosis in children.^84^ The second systematic review was a formal rating of guidelines^25^ published between 2014 and 2019 for treatment of latent and active tuberculosis.^85^ Guidelines from the World Health Organization,^86^ the United States,^28^ and the United Kingdom^87^ were rated as high-quality guidelines, though only the United States guideline had any recommendations for adverse event management and none of those recommendations were specific or had formal ratings of strength of evidence base.^28^

There is increasing evidence that adverse events are a major barrier to successful treatment. In a large clinical trial of adults receiving treatment for drug-sensitive pulmonary TB, participants who had a grade 3 or higher adverse event had a three-fold increased risk of having treatment failure, recurrence, or death during treatment,^2^ and cohort studies have shown similar increased risks.^5^^,^^15^ Notably, the relative risk of poor tuberculosis outcomes with intermittent dosing vs. daily dosing is comparable to that of adverse events,^88^^,^^89^ yet dosing frequency has been prominent subject of recent international and prominent national guidelines^28^^,^^33^^,^^74^ whilst adverse events have received little attention. Another example of the lack of attention on adverse event management is that the only substantive changes over the past 20 years in three of the most prominent guidelines was that adverse event management was removed from the WHO treatment guidelines^31^ and from the 2025 update to the U.S. guideline.^78^

We suspect that the lack of high-quality evidence regarding adverse event management during tuberculosis treatment has led to the lack of recommendations in prominent guidelines; only four of the many recommendations in our systematic review had formal ratings of the strength of the recommendation and evidence base. In informal discussions with colleagues who have been participants in recent tuberculosis treatment guidelines, we have heard the sentiment that making recommendations for adverse event management must wait until there is stronger data for such recommendations. We disagree as this leaves frontline clinicians and patients without management advice, even if based on expert opinion only, for common and serious treatment-related adverse events. Furthermore, failing to include recommendations for factors that have an important impact on treatment outcomes is contrary to the GRADE standards, a well-known set of recommendations for writing guidelines: “GRADE encourages panels to deal with their discomfort and to make recommendations even when confidence in effect estimate is low … Such recommendations will inevitably be weak, and may be accompanied by qualifications”.^90^ Given the lack of high-quality evidence for adverse event management, one might expect that treatment guidelines would highlight the need for studies in the field, yet only two guidelines included adverse event management as a research priority in improving tuberculosis treatment.^37^^,^^44^

The lack of high-quality data on adverse event management during drug sensitive tuberculosis treatment is a major gap in research. However, guidelines have also failed to take advantage of available data on key adverse events. For example, the epidemiology of adverse events during tuberculosis treatment has been studied for decades, yet very few guidelines included information on consistent risk factors for specific adverse events, such as the increased risk of drug induced liver injury with increasing age.^12^ Similarly, there are well-documented, clinically-significant interactions between rifampicin and several commonly-used medications to treat gastrointestinal adverse events (e.g., ondansetron, omeprazole)^79^, ^80^, ^81^ yet no guidelines included this information.

The example of nausea management provides another example of how available information has not been used in tuberculosis treatment guidelines. Despite the lack of clinical trials or cohort data on nausea management during tuberculosis treatment, there are detailed evidence-based guidelines for management of nausea during chemotherapy,^91^ radiation therapy,^92^ and following surgery.^93^ The consistency of the findings of clinical trials for nausea management in these three distinct clinical settings argues that their lessons should be the basis for designing research studies for management of nausea during tuberculosis treatment (e.g., that prevention is better than treating nausea; that serotonin receptor antagonists, such as ondansetron, are the initial drug of choice^92^, ^93^, ^94^; and that adding olanzapine is an evidence-based second-line intervention).^95^^,^^96^ We need both research on management of adverse events during tuberculosis treatment and a rigorous review of the available evidence from other fields of medicine.

To be effective in improving patient care, treatment guidelines should be quick and easy for busy front-line clinical care providers to use. Our review demonstrated notable gaps in ease of use. Only one of the international and prominent national guidelines included a table or algorithm regarding adverse event management,^28^ there was a notable lack of weblinks for more detailed information and specific recommendations for medications (drug name, dose) for managing common adverse events, and only one guideline had any recommendations for dosing of anti-emetic medications in children.^47^ On the positive side, some guidelines from non-governmental organizations demonstrated a number of user-friendly features: detailed review of risk factors for treatment-related adverse events,^44^ convenient web-based interactive guidelines with specific recommendations available in a few clicks,^45^^,^^47^ and detailed algorithms and nuanced recommendations for managing suspected drug-induced liver injury.^46^

Our systematic review has at least five limitations. First, we were unable to obtain guidelines from five of the high-burden countries and one guideline that was identified in the PubMed search. Second, we used an artificial intelligence program to translate 8 guidelines. We used a second artificial intelligence tool to evaluate translations by Chat GPT, but we did not have all translations reviewed by a native speaker of those languages, and we were unable to evaluate some aspects of “ease of use” in those guidelines. Third, we did not have all chart abstractions done by two independent reviewers, though we did do extensive checks of the data during the analysis. Fourth, we did not include review articles in our systematic review, and those articles may be a source for recommendations for adverse event management.^97^ Finally, we do not have information on the availability of laboratory testing and medications used to diagnose and treat adverse events in high-burden countries, a factor that may affect recommendations for adverse event management.

Guidelines should address all aspects of treatment that are associated with treatment outcomes, even when the available evidence is of low quality. Treatment-related adverse events decrease quality of life for patients,^6^ increase the risk of poor treatment outcomes, and require substantial resources of tuberculosis programs.^11^ Not all guidelines need to have recommendations for managing adverse events, but given their frequency and impact, guidelines should at least refer their users to detailed recommendations for management of common adverse events. Clearly, we need research to identify better-tolerated treatment regimens,^98^ but in parallel we also need research on how to manage the adverse events we face with current therapy. The cancer chemotherapy field has shown how better management of adverse events can enhance the patient experience with treatment and improve overall treatment outcomes. The prevention and management of nausea and vomiting for patients undergoing chemotherapy has been central to success in this area.^99^ There are important opportunities for us to learn from other fields of medicine to improve care and outcomes for our patients. Where recommendations do exist, there are opportunities to improve the presentation of recommendations for adverse event management. Greater use of clear labeling of sections on adverse events, tables and algorithms, and web-links can all make it easier for busy care providers to take care of their patients. Finally, mobile health Apps that provide quick and convenient access to recommendations for adverse event management could be an important way to get key recommendations to patients and frontline clinical staff.

## Contributors

All authors contributed to the design of the systematic review. WB performed the PubMed search, title, abstract and full text review, extracted data, and did data analysis. JE, GH, and KH identified and evaluated mobile health Apps, and collected and performed data abstraction on high-burden country guidelines. WB wrote the first draft of the paper. All authors contributed to the final draft of the paper and have read and approved the final version of the manuscript. All authors had full access to the data in the paper and verified the underlying data.

## Data sharing statement

The study protocol, search strategy, details of selection of treatment guidelines for inclusion, and the specific results for each of the treatment guidelines included in this systematic review are available in the on-line Supplementary Materials with this article. Inquiries about other data or materials from the study can be directed to Bill.burman@dhha.org.

## Declaration of interests

The authors declare no conflicts of interest. This research was supported by Wellcome Trust Clinical PhD Fellowship (Grant 203905/Z/16/Z to JE and Grant 223499/Z/21/Z to KH).
