# Supplementary material for: Adequacy of recommendations for adverse event management in national and international treatment guidelines for rifampicin-susceptible tuberculosis: a systematic review
Source: eClinicalMedicine. 2025 Mar 18;82:103148. doi: 10.1016/j.eclinm.2025.103148 (PMC11957802; doi:10.1016/j.eclinm.2025.103148)
Supplement: Supplementary Materials [file mmc1.docx]

**Supplementary materials**

| **Item** | **Pages** |
| --- | --- |
| Protocol for this systematic review | 2-5 |
| Supplementary Table 1. Details of the PubMed search for guidelines for treatment of active, rifampin-susceptible tuberculosis | 6 |
| Supplementary Table 2. Details of the internet search of treatment guideline repositories | 7 |
| Supplementary Table 3. Disposition of the articles selected for full-text review | 8-9 |
| Supplementary Table 4. Details of the search for mobile health Apps containing recommendations for adverse event management | 10 |
| Supplementary Table 5. Presence of recommendations for adverse event management: international and prominent country treatment guidelines | 11 |
| Supplementary Table 6. Presence of recommendations for adverse event management: non-governmental guidelines | 12 |
| Supplementary Table 7. Presence of recommendations for adverse event management: high-burden countries | 13 |
| Supplementary Table 8. Assessment of ease of use among guidelines that provided recommendations for managing adverse events | 14-15 |
| Supplementary Table 9. Changes in international guidelines for TB treatment over time: United States, European Union, World Health Organization | 16-17 |
| Supplementary Table 10. Details regarding recommendations for managing nausea/vomiting | 18-19 |
| Supplementary Table 11. Details regarding recommendations for managing suspected drug-induced liver injury | 20-21 |
| References for supplementary materials | 22-24 |

**Title:**

A systematic review of recommendations for adverse event prevention and management in national and international treatment guidelines for rifampin-susceptible TB

**Registration:**

We will register in PROSPERO

**Support for the systematic review:**

No outside support

**Rationale**

Treatment-related adverse events (AEs) are common ^1-4^ and quite problematic in the treatment of rifampin-susceptible TB using the DOTS regimen (isoniazid, rifampin, pyrazinamide, and ethambutol). Adverse events are associated with missed doses and treatment interruptions ^5-7^, regimen changes and extensions ^8,9^, failure to complete treatment^10^, and increases in treatment failure, recurrence, and death. ^1,4,11^ Moreover, some populations – elderly persons and persons with HIV co-infection ^12^, diabetes ^13^, and alcohol use disorder ^14^ – are at higher risk for drug intolerance and adverse events, translating to worse outcomes in programmatic settings. ^8,9^.

Common treatment-related AEs include nausea and vomiting, hepatotoxicity, skin rash, peripheral neuropathy, visual disturbances, and arthralgias ^2^. Despite their frequency and impacts on treatment outcomes, treatment-related AEs have not been a focus of clinical trials. As a result, patients and front-line care providers may have relatively little guidance about how to prevent and manage common AEs during the treatment of rifampin-susceptible TB. In contrast, AEs have been a major focus of research on new regimens to prevent TB and new regimens to treat rifampin-resistant TB.

During a recent course on TB research, we did an initial evaluation of recommendations for AE prevention and management in national and international TB treatment guidelines. We found very few recommendations in these guidelines (World Health Organization [WHO], United States, United Kingdom, European Union) and that the recommendations that were provided were usually based on expert opinion only.

Therefore, this is an opportune time to systematically review recommendations for AE prevention and management in national and international TB treatment guidelines. Identifying gaps in treatment guidelines may be useful in improving subsequent versions of those guidelines and in setting research priorities.

**Objectives**

1. To assess the recommendations made for preventing and managing common AEs in TB treatment guidelines for rifampin-susceptible TB of international organizations, the 30 countries that have the highest TB burden, and from selected countries whose guidelines are often used as a reference in other TB treatment guidelines (e.g., U.S. and WHO guidelines). Hypothesis – TB treatment guidelines provide little guidance to front-line clinicians and patients on ways to avoid and manage common AEs.
2. To evaluate the evidence base for recommendations for the prevention and management of AEs in international and national TB treatment guidelines. Hypothesis – Most recommendations are made based on expert opinion only.
3. To evaluate whether recommendations for AE prevention and management in WHO and U.S. guidelines have changed over the past 20 years. Hypothesis – There has been few changes in recommendations over this time period.
4. Evaluate the ease of use of these on-line guidelines. Hypothesis – Treatment guidelines may not be designed to promote ease of use and quick navigation through the website.

**Methods**

Eligibility criteria

Inclusion criteria

- - Governmental or non-governmental guidelines for treatment of active, rifampin-susceptible TB
    - Governmental guidelines – WHO, the 30 high TB burden countries (per the WHO TB 2023 report), United States, United Kingdom, European Union, and Australia. In addition to TB treatment guidelines, we will also review HIV treatment guidelines that include TB management recommendations (e.g., the United States Opportunistic Infections guidelines)
    - Non-governmental guidelines - Médecins Sans Frontières (MSF), Up-to-Date

Exclusion criteria

- - Guidelines for treatment to prevent TB
  - Guidelines for treatment for treatment of rifampin-resistant TB

Information sources:

- PubMed
- Websites of the governmental and non-governmental entities above – we will use the latest set of TB treatment guidelines, if multiple versions are available
- International registries of guidelines
- References from papers identified in PubMed and from the websites, as above
- Apps created to provide guidance to front-line providers
- To evaluate changes over time in recommendations, we will compare current TB treatment guidelines from WHO, the United States, and the European Union to versions from approximately 20 years ago.

Search strategy

- Search terms – tuberculosis; adverse event, side effects, guidelines

**Study records**

Data management

- EndNotes software package will be used to manage the review the output of the search

Selection process

- One reviewer will evaluate titles from the literature search
- One reviewer will evaluate the full-text papers identified as above

Data collection process

- We will try to contact staff at Ministries of Health if we are unable to find TB treatment guidelines from the governments included in the list above.
- Guidelines published in languages other than English will be translated into English using Chat GPT

Guideline quality assessment regarding prevention and management of treatment-related adverse events

- The three measures in the “Clarity of presentation” section of the AGREE II instrument (<https://www.agreetrust.org/wp-content/uploads/2017/12/AGREE-II-Users-Manual-and-23-item-Instrument-2009-Update-2017.pdf>) will be used to assess the quality of sections on adverse event prevention and management, for guidelines other than those from the 30 high-burden countries

Data items

|  | Data element | Comment |
| --- | --- | --- |
| Source of the guidelines | Country or organization |  |
| Name of the guidelines |  |  |
| Year | Year issued or updated |  |
| Overview | Number of tables and figures devoted to recommendations on AEs |  |
|  | Was evidence of the frequency of adverse events reviewed? |  |
|  | Were risk demographic and clinical risk factors for adverse events reviewed? |  |
| Common AEs | Recommendations for prevention and management of nausea/vomiting | Not present, summary of the recommendations made |
|  | - Description of non-pharmacologic and pharmacologic measures for nausea recommended |  |
|  | - If present, the evidence base for the recommendations | Randomized trials, cohort studies, expert opinion |
|  | Recommendations for prevention and management of hepatotoxicity | Not present, summary of the recommendations made |
|  | - If present, are there recommendations for when to stop therapy? | Randomized trials, cohort studies, expert opinion |
|  | - Recommendations for when to re-start therapy? |  |
|  | - Recommendations for how to re-start therapy (sequentially, other). If so, what order of restart is recommended? |  |
|  | Recommendations for prevention and management of skin rash | Not present, summary of the recommendations made |
|  | - If present, the evidence base for the recommendations | Randomized trials, cohort studies, expert opinion |
|  | Recommendations for prevention and management of peripheral neuropathy | Not present, summary of the recommendations made |
|  | - If present, the evidence base for the recommendations | Randomized trials, cohort studies, expert opinion |
|  | Recommendations for prevention and management of visual disturbances | Not present, summary of the recommendations made |
|  | - If present, the evidence base for the recommendations | Randomized trials, cohort studies, expert opinion |
|  | Recommendations for prevention and management of arthralgias | Not present, summary of the recommendations made |
|  | - If present, the evidence base for the recommendations | Randomized trials, cohort studies, expert opinion |
| Ease of use | Searchable on-line resource |  |
|  | Links in the on-line resource functional |  |
|  | Presence of tables, bulleted lists, algorithms |  |
|  | Three measures from the AGREE II instrument, assessed for guidelines other than the 30 high-burden countries | - Specific and unambiguous recommendations - Different options presented - Key recommendations easily identifiable |
|  | Overall comments on ease of use |  |
| Comments | Any other comments that come from the review |  |

**Planned analyses**

- Number of guidelines that include recommendations for preventing and managing treatment-related AEs
- AEs for which recommendations are provided (% for each major category of AE)
- Of the recommendations made, evidence base used (% based on randomized trials, % based on cohort studies, % based on expert advice
- Summary of the changes made over the past 20 years in guidelines from the WHO, United States, and European Union
- Assessment of the ease of use of the recommendations using the score from the AGREE II domain on “Clarity of presentation” and qualitative comments of the reviewers

**References**

1. Tweed CD, Crook AM, Amukoye EI, et al. Toxicity associated with tuberculosis chemotherapy in the REMoxTB study. *BMC Infect Dis*. Jul 11 2018;18(1):317. doi:10.1186/s12879-018-3230-6

2. Marra F, Marra CA, Bruchet N, et al. Adverse drug reactions associated with first-line anti-tuberculosis drug regimens. *Int J Tuberc Lung Dis*. Aug 2007;11(8):868-75.

3. Lorent N, Sebatunzi O, Mukeshimana G, Van den Ende J, Clerinx J. Incidence and risk factors of serious adverse events during antituberculous treatment in Rwanda: a prospective cohort study. *PLoS One*. 2011;6(5):e19566. doi:10.1371/journal.pone.0019566

4. Lv X, Tang S, Xia Y, et al. Adverse reactions due to directly observed treatment strategy therapy in Chinese tuberculosis patients: a prospective study. *PLoS One*. 2013;8(6):e65037. doi:10.1371/journal.pone.0065037

5. Dixon EG, Rasool S, Otaalo B, et al. No action is without its side effects: Adverse drug reactions and missed doses of antituberculosis therapy, a scoping review. *Br J Clin Pharmacol*. Jan 2024;90(1):313-320. doi:10.1111/bcp.15908

6. Oh AL, Makmor-Bakry M, Islahudin F, Wong IC. Prevalence and predictive factors of tuberculosis treatment interruption in the Asia region: a systematic review and meta-analysis. *BMJ Glob Health*. Jan 2023;8(1)doi:10.1136/bmjgh-2022-010592

7. Fox WS, Strydom N, Imperial MZ, Jarlsberg L, Savic RM. Examining nonadherence in the treatment of tuberculosis: The patterns that lead to failure. *Br J Clin Pharmacol*. Jul 2023;89(7):1965-1977. doi:10.1111/bcp.15515

8. Louie JK, Keh C, Agraz-Lara R, Phillips A, Graves S. Adverse Events Associated With Treatment for Pan-Susceptible Tuberculosis in San Francisco. *Clin Infect Dis*. Mar 21 2023;76(6):1121-1124. doi:10.1093/cid/ciac867

9. Kwon BS, Kim Y, Lee SH, et al. The high incidence of severe adverse events due to pyrazinamide in elderly patients with tuberculosis. *PLoS One*. 2020;15(7):e0236109. doi:10.1371/journal.pone.0236109

10. Cardoso MA, do Brasil P, Schmaltz CAS, Sant'Anna FM, Rolla VC. Tuberculosis Treatment Outcomes and Factors Associated with Each of Them in a Cohort Followed Up between 2010 and 2014. *Biomed Res Int*. 2017;2017:3974651. doi:10.1155/2017/3974651

11. Shang P, Xia Y, Liu F, et al. Incidence, clinical features and impact on anti-tuberculosis treatment of anti-tuberculosis drug induced liver injury (ATLI) in China. *PLoS One*. 2011;6(7):e21836. doi:10.1371/journal.pone.0021836

12. Breen RA, Miller RF, Gorsuch T, et al. Adverse events and treatment interruption in tuberculosis patients with and without HIV co-infection. *Thorax*. Sep 2006;61(9):791-4. doi:10.1136/thx.2006.058867

13. Siddiqui AN, Khayyam KU, Sharma M. Effect of Diabetes Mellitus on Tuberculosis Treatment Outcome and Adverse Reactions in Patients Receiving Directly Observed Treatment Strategy in India: A Prospective Study. *Biomed Res Int*. 2016;2016:7273935. doi:10.1155/2016/7273935

14. Przybylski G, Dabrowska A, Trzcinska H. Alcoholism and other socio-demographic risk factors for adverse TB-drug reactions and unsuccessful tuberculosis treatment - data from ten years' observation at the Regional Centre of Pulmonology, Bydgoszcz, Poland. *Med Sci Monit*. Mar 19 2014;20:444-53. doi:10.12659/MSM.890012

**Supplementary Table 1. Details of the PubMed search for treatment of active, rifampin-susceptible tuberculosis**

| Number | Search | Records |
| --- | --- | --- |
| 1 | Guideline or guidelines | 67,019 |
| 2 | Tuberculosis | 6,194 |
| 2 | Limit to past 20 years | 5,064 |

**Supplementary Table 2. Details of the internet search of treatment guideline repositories**

| Guideline repository | URL |
| --- | --- |
| PREPARE (Practice guideline registration for transparency) | <http://www.guidelines-registry.org/> |
| GIN (Guidelines International Network) | <https://g-i-n.net/international-guidelines-library> |
| Guideline Central | <https://www.guidelinecentral.com/> |
| Grading of Recommendations Assessment, Development and Evaluation (GRADEpro GDT) | <https://guidelines.gradepro.org/search/%22Tuberculosis%20of%20lung%2C%20confirmed%20by%20sputum%20microscopy%20with%20or%20without%20culture%22?type=_all> |
| Agency for Healthcare Research and Quality | <https://www.ahrq.gov/prevention/guidelines/index.html> |
| Médecins Sans Frontières / Doctors Without Borders | <https://medicalguidelines.msf.org/en> |
| Emergency Care Research Institute (ECRI) | <https://guidelines.ecri.org/> |
| National Institute for Health and Care Excellence (NICE) | <https://www.nice.org.uk/> |
| Canadian Agency for Drugs and Technology for Health (CADTH) | <https://www.cda-amc.ca/search?s=> |
| Canadian review of TB treatment guidelines | <https://www.cda-amc.ca/treatment-tuberculosis-review-guidelines> |
| University of California at San Francisco Center for Tuberculosis | <https://tb.ucsf.edu/policy-guidelines> |
| European Centre for Disease Prevention and Control | <https://www.ecdc.europa.eu/en/all-topics-z/tuberculosis/prevention-and-control/european-union-standards-tuberculosis-care> |
| Treatment Action Group, Global TB Community Advisory Board * | <https://www.tbonline.info/guidelines/> |

* The Treatment Action Group’s Global TB Community Advisory Board (<https://www.tbonline.info/guidelines/>) had the most complete listing of tuberculosis treatment guidelines

**Supplementary Table 3. Disposition of the articles selected for full-text review**

|  | Included | Reason for not including |
| --- | --- | --- |
| American Thoracic Society/Centers for Disease Control and Prevention/Infectious Diseases Society of America: controlling tuberculosis in the United States. Am J Respir Crit Care Med. 2005;172(9):1169-227. doi: 10.1164/rccm.2508001. PubMed PMID: 16249321. | Yes |  |
| API TB Consensus Guidelines 2006: Management of pulmonary tuberculosis, extra-pulmonary tuberculosis and tuberculosis in special situations. J Assoc Physicians India. 2006;54:219-34. PubMed PMID: 16800350. | No | More recent guidelines |
| Guidance for national tuberculosis programmes on the management of tuberculosis in children. Chapter 2: anti-tuberculosis treatment in children. Int J Tuberc Lung Dis. 2006;10(11):1205-11. PubMed PMID: 17131777. | Yes |  |
| National Institute for Health and Care Excellence: Guidelines. Tuberculosis. London: National Institute for Health and Care Excellence (NICE) | Yes |  |
| Expert consensus on the treatment of drug susceptible tuberculosis in children and adolescents. Chinese Journal of Applied Clinical Pediatrics. 2024;39(9):641-5. doi: 10.3760/cma.j.cn101070-20240529-00334. | No | Unable to obtain a copy |
| Akkerman OW, Duarte R, Tiberi S, Schaaf HS, Lange C, Alffenaar JWC, et al. Clinical standards for drug-susceptible pulmonary TB. Int J Tuberc Lung Dis. 2022;26(7):592-604. doi: 10.5588/ijtld.22.0228. PubMed PMID: 35768923; PubMed Central PMCID: PMCPMC9272737. | No | Not a general guideline for tuberculosis treatment |
| Blumberg HM, Leonard MK, Jr., Jasmer RM. Update on the treatment of tuberculosis and latent tuberculosis infection. Jama. 2005;293(22):2776-84. doi: 10.1001/jama.293.22.2776. PubMed PMID: 15941808. | No | Not a general guideline for tuberculosis treatment |
| Bracchi M, van Halsema C, Post F, Awosusi F, Barbour A, Bradley S, et al. British HIV Association guidelines for the management of tuberculosis in adults living with HIV 2019. HIV Med. 2019;20 Suppl 6:s2-s83. doi: 10.1111/hiv.12748. PubMed PMID: 31152481. | Yes |  |
| Carr W, Kurbatova E, Starks A, Goswami N, Allen L, Winston C. Interim Guidance: 4-Month Rifapentine-Moxifloxacin Regimen for the Treatment of Drug-Susceptible Pulmonary Tuberculosis - United States, 2022. MMWR Morb Mortal Wkly Rep. 2022;71(8):285-9. Epub 20220225. doi: 10.15585/mmwr.mm7108a1. PubMed PMID: 35202353. | No | Not a general guideline for tuberculosis treatment |
| Chiang SS, Graham SM, Schaaf HS, Marais BJ, Sant'Anna CC, Sharma S, et al. Clinical standards for drug-susceptible TB in children and adolescents. Int J Tuberc Lung Dis. 2023;27(8):584-98. doi: 10.5588/ijtld.23.0085. PubMed PMID: 37491754; PubMed Central PMCID: PMCPMC10365562. | No | Not a general guideline for tuberculosis treatment |
| Conde MB, Melo FA, Marques AM, Cardoso NC, Pinheiro VG, Dalcin Pde T, et al. III Brazilian Thoracic Association Guidelines on tuberculosis. J Bras Pneumol. 2009;35(10):1018-48. doi: 10.1590/s1806-37132009001000011. PubMed PMID: 19918635. | Yes |  |
| Hopewell PC, Pai M, Maher D, Uplekar M, Raviglione MC. International standards for tuberculosis care. Lancet Infect Dis. 2006;6(11):710-25. doi: 10.1016/s1473-3099(06)70628-4. PubMed PMID: 17067920. | Yes |  |
| Migliori GB, Sotgiu G, Rosales-Klintz S, Centis R, D'Ambrosio L, Abubakar I, et al. ERS/ECDC Statement: European Union standards for tuberculosis care, 2017 update. Eur Respir J. 2018;51(5). Epub 20180517. doi: 10.1183/13993003.02678-2017. PubMed PMID: 29678945. | Yes |  |
| Migliori GB, Zellweger JP, Abubakar I, Ibraim E, Caminero JA, De Vries G, et al. European union standards for tuberculosis care. Eur Respir J. 2012;39(4):807-19. doi: 10.1183/09031936.00203811. PubMed PMID: 22467723; PubMed Central PMCID: PMCPMC3393116. | Yes |  |
| Milburn H, Ashman N, Davies P, Doffman S, Drobniewski F, Khoo S, et al. Guidelines for the prevention and management of Mycobacterium tuberculosis infection and disease in adult patients with chronic kidney disease. Thorax. 2010;65(6):557-70. doi: 10.1136/thx.2009.133173. PubMed PMID: 20522863. | Yes |  |
| Nahid P, Dorman SE, Alipanah N, Barry PM, Brozek JL, Cattamanchi A, et al. Official American Thoracic Society/Centers for Disease Control and Prevention/Infectious Diseases Society of America Clinical Practice Guidelines: Treatment of Drug-Susceptible Tuberculosis. Clin Infect Dis. 2016;63(7):e147-e95. Epub 20160810. doi: 10.1093/cid/ciw376. PubMed PMID: 27516382; PubMed Central PMCID: PMCPMC6590850. | Yes |  |
| Thwaites G, Fisher M, Hemingway C, Scott G, Solomon T, Innes J. British Infection Society guidelines for the diagnosis and treatment of tuberculosis of the central nervous system in adults and children. J Infect. 2009;59(3):167-87. Epub 20090704. doi: 10.1016/j.jinf.2009.06.011. PubMed PMID: 19643501. | Yes |  |

**Supplementary Table 4. Details of the search for mobile health Apps containing recommendations for adverse event management**

| Name of mobile health application | Source | Accessible from the United Kingdom | Accessible from the United States of America | Accessible from Uganda | Adverse event recommendations included |
| --- | --- | --- | --- | --- | --- |
| TB HDL | Apple store | No | No | No | No |
| Georgia TB Reference Guide | Google App store | Yes | Yes | Yes | No |
| LTBI Care | Literature review * | No | No | No | No |
| EasyTB | Literature review | No | No | No | No |
| Diagnosa Tuberkulosis (TB) | Literature review | No | No | No | No |
| TB Clinical guide | Literature review | No | No | No | No |
| SNTC | Literature review | No | No | No | No |
| TBeReview HIVTB | Literature review | No | No | No | No |
| TBeReview DRTB | Literature review | No | No | No | No |
| Medical Management of MDR-TB | Literature review | No | No | No | No |
| TB Doctor | Literature review | No | No | No | No |
| eTB Manager | ChatGPT | No | No | No | No |
| TUBER | ChatGPT | No | No | No | No |
| TB Mobile | ChatGPT | No | No | No | No |
| CommTB | Google App store | No | No | No | No |
| Manage TB India | Google App store | No | No | No | No |
| Explain TB | Apple store | Yes | Yes | No | No |
| TB Companion | Apple store | Yes | Yes | No | No |
| WHO TB guide | Apple store | Yes | Yes | Yes | No |
| IDSA Practice Guidelines | Apple store | Yes | Yes | Yes | Yes † |
| MSF treatment guidelines | Apple store | Yes | Yes | Yes | Yes † |

* Apps listed in a prior publication on mobile health resources for tuberculosis care ^1^

† App connected to the on-line version of the guideline that was already included in the systematic review

**Supplementary Table 5. Presence of recommendations for adverse event management: international and prominent country treatment guidelines**

| Name of the guideline | Adverse event management | Nausea and/or vomiting | Hepatotox-icity | Skin reactions | Neuropathy | Visual changes | Drug fever | Arthralgia |
| --- | --- | --- | --- | --- | --- | --- | --- | --- |
| Stop TB Partnership: Anti-tuberculosis treatment in children ^2^ | Present | Not present | Present | Not present | Present | Not present | Not present | Not present |
| International standards for tuberculosis Care ^3^ | Not present |  |  |  |  |  |  |  |
| United States (ATS/CDC/IDSA) Guidelines for Treatment of Drug-Susceptible Tuberculosis ^4^ | Present | Present | Present | Present | Not present | Present | Present | Not present |
| European Union Standards for Tuberculosis Care ^5^ | Not present |  |  |  |  |  |  |  |
| WHO: drug-susceptible tuberculosis treatment ^6^ | Not present |  |  |  |  |  |  |  |
| WHO: treatment for isoniazid resistant tuberculosis ^7^ | Not present |  |  |  |  |  |  |  |
| WHO: tuberculosis in children and adolescents ^8^ | Not present |  |  |  |  |  |  |  |
| Canadian Tuberculosis Standards ^9^ | Not present |  |  |  |  |  |  |  |
| Australian National Guidelines ^10^ | Not present |  |  |  |  |  |  |  |
| Spanish guidelines for antiretroviral therapy ^11^ | Not present |  |  |  |  |  |  |  |
| United States. Guidelines for the Prevention and Treatment of Opportunistic Infections ^12^ | Present | Not present | Present | Present | Not present | Not present | Not present | Not present |
| United Kingdom (NICE) – Tuberculosis ^13^ | Present | Not present | Present | Not present | Not present | Not present | Not present | Not present |

**Supplementary Table 6. Presence of recommendations for adverse event management: non-governmental guidelines**

| Name of the guideline | Adverse event management | Nausea and/or vomiting | Hepatotox-icity | Skin reactions | Neuropathy | Visual changes | Drug fever | Arthralgia |
| --- | --- | --- | --- | --- | --- | --- | --- | --- |
| British Infection Society: diagnosis and treatment of tuberculosis of the central nervous system ^14^ | Present | Not present | Present | Not present | Not present | Not present | Not present | Not present |
| British Thoracic Society: management of tuberculosis in patients with chronic kidney disease ^15^ | Not present |  |  |  |  |  |  |  |
| Spanish guidelines for diagnosis, treatment and prevention of tuberculosis ^16^ | Present | Present | Present | Present | Present | Not present | Present | Not present |
| IUATLD management of tuberculosis: A Guide to Essential Practice ^17^ | Present | Not present | Present | Not present | Not present | Present | Not present | Not present |
| European AIDS Clinical Society Guidelines ^18^ | Not present |  |  |  |  |  |  |  |
| British HIV Association guidelines for the management of tuberculosis in adults living with HIV ^19^ | Present | Present | Present | Present | Present | Not present | Not present | Not present |
| Clinical standards for the management of adverse effects during treatment for TB ^20^ | Present | Present | Present | Present | Present | Present | Present | Not present |
| Treatment of drug-susceptible tuberculosis in non-pregnant adults without HIV infection ^21^ | Present | Present | Present | Present | Present | Present | Not present | Present |
| Management of drug-induced liver injury in people with HIV treated for tuberculosis: 2024 update (South Africa) ^22^ | Present | Not present | Present | Not present | Not present | Not present | Not present | Not present |
| MSF - Tuberculosis: Practical guide for clinicians, nurses, laboratory technicians and medical auxiliaries ^23^ | Present | Present | Present | Present | Present | Present | Not present | Present |

IUATLD – International Union Against Tuberculosis and Lung Disease

MSF - Médecins Sans Frontières

**Supplementary Table 7. Presence of recommendations for adverse event management: high-burden countries**

| Name of the guideline | Adverse event management | Nausea and/or vomiting | Hepatotox-icity | Skin reactions | Neuropathy | Visual changes | Drug fever | Arthralgia |
| --- | --- | --- | --- | --- | --- | --- | --- | --- |
| Bangladesh ^24^ | Present | Present | Present | Present | Present | Present | Not present | Present |
| Brazil ^25^ | Present | Not present | Present | Not present | Not present | Not present | Not present | Not present |
| China ^26^ | Present | Present | Present | Present | Present | Present | Present | Present |
| Congo (Democratic Republic) | Present | Present | Present | Present | Present | Present | Present | Present |
| Ethiopia ^27^ | Present | Present | Present | Present | Present | Present | Not present | Not present |
| India ^28^ | Not present |  |  |  |  |  |  |  |
| Indonesia | Present | Not present | Present | Not present | Present | Present | Present | Not present |
| Kenya ^29^ | Present | Present | Present | Present | Present | Present | Not present | Present |
| Korea ^30^ | Present | Present | Present | Present | Present | Present | Present | Present |
| Lesotho ^31^ | Present | Present | Present | Present | Present | Present | Present | Present |
| Liberia ^32^ | Present | Present | Present | Present | Present | Present | Not present | Present |
| Mongolia ^33^ | Not present |  |  |  |  |  |  |  |
| Mozambique ^34^ | Present | Present | Present | Present | Present | Present | Present | Present |
| Myanmar ^35^ | Present | Present | Present | Present | Present | Present | Not present | Present |
| Namibia ^36^ | Present | Present | Present | Present | Present | Present | Present | Present |
| Nigeria ^37^ | Present | Present | Present | Present | Present | Present | Not present | Present |
| Pakistan ^38^ | Present | Present | Present | Present | Present | Present | Present | Present |
| Philippines ^39^ | Present | Present | Present | Present | Present | Present | Not present | Present |
| Sierra Leone ^40^ | Present | Present | Present | Present | Present | Present | Not present | Present |
| South Africa ^41^ | Present | Not present | Present | Present |  | Present | Present | Present |
| Tanzania ^42^ | Present | Present | Present | Present | Present | Present | Not present | Present |
| Thailand ^43^ | Present | Present | Present | Present | Present | Present | Present | Present |
| Uganda ^44^ | Present | Present | Present | Present | Present | Present | Not present | Present |
| Vietnam ^45^ | Present | Not present | Present | Not present | Present | Present | Present | Present |
| Zambia ^46^ | Present | Present | Present | Not present | Present | Present | Not present | Not present |

**Supplementary Table 8. Assessment of ease of use among guidelines that provided recommendations for managing adverse events**

| Name of the guideline | Review of risk factors for adverse events | Number of tables/algorithms for adverse events | Ease of navigation to adverse event recommendations | Functional weblinks | Specific medications | Specific dose |
| --- | --- | --- | --- | --- | --- | --- |
| Stop TB Partnership: Anti-tuberculosis treatment in children ^2^ | No | 0 | Yes | No | No |  |
| United States (ATS/CDC/IDSA) Guidelines for Treatment of Drug-Susceptible Tuberculosis ^4^ | No | 1 | No * | No | No |  |
| Guidelines for the Prevention and Treatment of Opportunistic Infections in Adults and Adolescents With HIV (United States) ^12^ | No | 0 | Yes | Yes | No |  |
| United Kingdom (NICE) – Tuberculosis ^13^ | No | 0 | Yes | Yes | No |  |
| British Infection Society guidelines for the diagnosis and treatment of tuberculosis of the central nervous system in adults and children ^14^ | No | 1 | Yes | No | No |  |
| Spanish guidelines for diagnosis, treatment and prevention of tuberculosis ^16^ | No | 1 | Yes | No | Yes | No |
| IUATLD management of tuberculosis: A Guide to Essential Practice ^17^ | No | 0 | Yes | No | No |  |
| British HIV Association guidelines for the management of tuberculosis in adults living with HIV ^19^ | No | 1 | Yes | Yes | Yes | Yes, B6 only |
| Clinical standards for the management of adverse effects during treatment for TB ^20^ | Yes | 6 | Yes | No | No |  |
| Up-to-Date: Treatment of drug-susceptible tuberculosis in non-pregnant adults without HIV infection ^21^ | No | 2 | Yes | Yes | No |  |
| Management of drug-induced liver injury in people with HIV treated for tuberculosis: 2024 update (South Africa) ^22^ | Yes | 4 | Yes | Yes | No |  |
| MSF Medical Guidelines, Tuberculosis: Practical guide for clinicians, nurses, laboratory technicians and medical auxiliaries ^23^ | No | 8 | Yes | Yes | Yes | Yes |
| Bangladesh ^24^ | Yes | 1 | Yes | No | Yes, B6 only | No |
| Brazil ^25^ | No | 0 | No | No | No |  |
| China ^26^ | Yes | † | | | Yes, B6 only | No |
| Congo (Democratic Republic) ^47^ | Yes |  | | | Yes, B6 only | No |
| Ethiopia ^27^ | Yes | 3 | Yes | No | Yes, B6 only | Yes, B6 only |
| Indonesia ^48^ | Yes | † | | | Yes, B6 only | No |
| Kenya ^29^ | Yes | 9 | Yes | No | Yes, B6 only | Yes, B6 only |
| Korea ^30^ | Yes | † | | | Yes | No |
| Liberia ^32^ | Yes | 2 | Yes | No | Yes, B6 only | Yes, B6 only |
| Lesotho ^31^ | Yes | 2 | Yes | No | Yes, B6 only | Yes, B6 only |
| Mozambique ^34^ | Yes | † | | | Yes | Yes |
| Myanmar ^35^ | No | 1 | Yes | No | Yes, B6 only | Yes, B6 only |
| Namibia ^36^ | Yes | 1 | Yes | No | Yes, B6 only | Yes, B6 only |
| Nigeria ^37^ | No | 1 | No | No | Yes, B6 only | Yes, B6 only |
| Pakistan ^38^ | No | 1 | Yes | No | Yes | Yes |
| Philippines ^39^ | No | 2 | Yes | No | Yes | Yes |
| Sierra Leone ^40^ | No | 2 | Yes | No | Yes, B6 only | Yes, B6 only |
| South Africa ^41^ | No | 2 | Yes | No | Yes, B6 only | Yes, B6 only |
| Tanzania ^42^ | No | 1 | Yes | No | Yes, B6 only | No |
| Thailand ^43^ | Yes | † | | | Yes, B6 only | Yes, B6 only |
| Uganda ^44^ | No | 1 | Yes | No | Yes, B6 only | Yes, B6 only |
| Vietnam ^45^ | Yes | † | | | Yes, B6 only | Yes, B6 only |
| Zambia ^46^ | Yes | 4 | Yes | No | Yes, B6 only | Yes, B6 only |

* Adverse event section under the heading "Practical aspects of treatment"

† Unable to assess using documents translated by ChatGPT

ATS/CDC/IDSA – American Thoracic Society, Centers for Disease Control and Prevention, Infectious Diseases Society of America

NICE – National Institute for Health and Care Excellence

IUATLD – International Union Against Tuberculosis and Lung Disease

MSF – Médecins Sans Frontières

B6 – pyridoxine (Vitamin B6)

**Supplementary Table 9. Changes in international guidelines for TB treatment over time: United States, European Union, World Health Organization**

| *United States guidelines (American Thoracic Society, Centers for Disease Control and Prevention, Infectious Diseases Society of America)* | | |
| --- | --- | --- |
|  | 2003 ^49^ | 2016 ^4^ |
| Gastrointestinal upset | Dosing with food | Dosing with light snack (low fat) |
|  |  | Antacids |
|  |  | Some experts suggest trial of a proton pump inhibitor |
|  |  | Caution about co-administration of divalent cations and fluoroquinolones |
| Rash | Antihistamine if rash is minor | Antihistamine if no fever or mucous membrane symptoms |
|  |  | Consider re-challenge in an inpatient setting |
|  |  | Corticosteroids can be used for systemic symptoms |
| Hepatotoxicity | Generally prudent to treat with three non-hepatotoxic drugs because of the time for AST to decrease | If hepatitis was severe and INH and RIF are tolerated, don’t re-challenge with PZA |
| Optic neuritis | Not discussed | Association with EMB |
|  |  | Usually after one month of therapy |
|  |  | Screen with visual acuity and color vision testing |
|  |  | Stop EMB if suspected |
|  |  |  |
| *European Union standards for tuberculosis care* | | |
|  | 2012 ^50^ | 2017 ^5^ |
|  | No mention of treatment-related adverse events | No mention of treatment-related adverse events |
|  |  |  |
| *World Health Organization TB treatment guidelines* | | |
|  | 2010 ^51^ | 2022 ^52^ |
| Introduction of the Section 4.9 “Monitoring and recording adverse events” | “Most TB patients complete their treatment without any significant adverse drug effect. However, a few patients do experience adverse events.” | No mention of treatment-related adverse events (Of note, this review included overall TB treatment guidelines, as well as guidelines on “TB Care and Support” and “Management of TB in children and adolescents”. |
| Rash | For itching without rash, try symptomatic treatment with antihistamines and skin moisturizing while continuing TB treatment |  |
|  | If rash develops stop all anti-TB drugs |  |
|  | When rash has resolved, sequential rechallenge with dose escalation, starting with IRF or INH |  |
| Drug-induced hepatitis | Note that TB therapy includes three drugs (INH, RIF, PZA) that can cause drug-induced liver disease |  |
|  | If hepatitis thought to be due to anti-TB drugs, all drugs should be stopped |  |
|  | If severely ill with TB, use a non-hepatotoxic regimen of STREP, EMB, and a fluoroquinolone |  |
|  | Wait for symptom resolution and liver function tests normalization |  |
|  | Sequential rechallenge: RIF, then INH, then PZA |  |
|  | If the patient had jaundice and INH and RIF are tolerated, don’t re-challenge with PZA |  |
|  | If only fixed-dose combinations are available – re-challenge with combination of HRE and STREP (if during the intensive phase). If during the continuation phase, rechallenge with HR |  |

**Supplementary Table 10. Details regarding recommendations for managing nausea/vomiting, among guidelines that provided recommendations for managing nausea/vomiting**

| Name of the guideline (reference) | Non-pharmacologic measures recommended | Medication class recommended | Specific medications recommended | Specific dose | Drug-drug interactions |
| --- | --- | --- | --- | --- | --- |
| United States (ATS/CDC/IDSA) Guidelines for Treatment of Drug-Susceptible Tuberculosis ^4^ | Dose with food, dose at bedtime | Antacids, proton pump inhibitor | None |  |  |
| Spanish guidelines for diagnosis, treatment and prevention of tuberculosis ^16^ | Split dose | Anti-emetics | Metoclopramide, omeprazole | No | No |
| British HIV Association guidelines for the management of tuberculosis in adults living with HIV ^19^ | Dose with food, split dose | Anti-emetics | No |  | No |
| Clinical standards for the management of adverse effects during treatment for TB ^20^ | Change time of dose, sit upright after dose | Anti-emetics | No |  | No |
| Up-to-Date: Treatment of drug-susceptible tuberculosis in non-pregnant adults without HIV infection ^21^ | Dose with food, split dose | Anti-emetics, proton pump inhibitor, antacids | No |  | Decreased absorption of fluoroquinolones with antacids |
| MSF Medical Guidelines, Tuberculosis: Practical guide for clinicians, nurses, laboratory technicians and medical auxiliaries ^23^ | Dose at bedtime | Anti-emetics, benzodiazepine | Ondansetron, metoclopramide, promethazine, diazepam | Yes | No |
| Bangladesh ^24^ | Dose with food | Antacids, proton pump inhibitor | No |  |  |
| China ^26^ | Dose with food | Anti-emetics | No |  |  |
| Congo (Democratic Republic of Congo) ^47^ | Dose with food | Anti-emetics | No |  |  |
| Ethiopia ^27^ | Dose with food |  |  |  |  |
| Kenya ^29^ | Dose with food, dose at bedtime, take dose slowly, avoid alcohol | Antacids | No |  |  |
| Korea (Republic of Korea) ^30^ | Dose with food | Anti-emetics | No |  |  |
| Lesotho ^31^ | Dose with food, dose at bedtime, take dose slowly |  |  |  |  |
| Liberia ^32^ | Dose with food, dose at bedtime, take dose slowly | Antacids | No | No | No |
| Mozambique ^34^ | Dose with food | Anti-emetics | Metoclopramide, ondansetron |  | Ondansetron and drugs that prolong QT |
| Myanmar ^35^ | Dose with food, dose at bedtime |  |  |  |  |
| Namibia ^36^ | Dose with food, dose at bedtime, take dose slowly |  |  |  |  |
| Nigeria ^37^ | Dose with food, avoid fatty and spicy foods |  |  |  |  |
| Pakistan ^38^ | Dose with food, dose at bedtime, take dose slowly | Anti-emetics, benzodiazepine | Metoclopramide, ondansetron, diazepam | Yes | Ondansetron and drugs that prolong QT |
| Philippines ^39^ | Dose with food | Anti-emetics | Metoclopramide, ondansetron, diazepam | Yes | No |
| Sierra Leone ^40^ | Dose with food, dose at bedtime, take dose slowly |  |  |  |  |
| Tanzania ^42^ | Dose with food, dose at bedtime, take dose slowly |  |  |  |  |
| Thailand ^43^ | Eat certain foods, drink plenty of water | Anti-emetics | No |  |  |
| Uganda ^44^ | Dose with food, dose at bedtime |  |  |  |  |
| Zambia ^46^ | Dose with food, dose at bedtime |  |  |  |  |

ATS/CDC/IDSA – American Thoracic Society, Centers for Disease Control and Prevention, Infectious Diseases Society of America

MSF – Médecins Sans Frontières

**Supplementary Table 11. Details regarding recommendations for managing suspected hepatotoxicity, among guidelines that provided recommendations for managing hepatoxicity**

| Name of the guideline | Criteria for stopping therapy | Type of re-challenge | PZA Rechallenge |
| --- | --- | --- | --- |
| Stop TB Partnership: Anti-tuberculosis treatment in children ^2^ | AST > 5 X ULN if asymptomatic | Not stated | Not stated |
| United States (ATS/CDC/IDSA) Guidelines for Treatment of Drug-Susceptible Tuberculosis ^4^ | AST > 3 X ULN with symptoms or > 5 X ULN without symptoms | Sequential | Consider |
| Guidelines for the Prevention and Treatment of Opportunistic Infections in Adults and Adolescents With HIV ^12^ | ALT >3 x ULN with symptoms; ALT > 3 X ULN and bilirubin > 2 X ULN without symptoms; or ALT > 5 X ULN without symptoms | Sequential | Consider |
| United Kingdom (NICE) – Tuberculosis ^13^ | Not stated | Sequential | No |
| British Infection Society guidelines for the diagnosis and treatment of tuberculosis of the central nervous system in adults and children ^14^ | AST/ALT > 5 x ULN - stop PZA and continue INH + RIF; if ALT continues to rise, stop INH and RIF | Dose escalation, sequential | Consider |
| Spanish guidelines for diagnosis, treatment and prevention of tuberculosis ^16^ | ALT > 3 x ULN with symptoms or ALT > 5 X ULN or alkaline phosphatase > 3 x ULN without symptoms | Dose escalation, sequential | Not stated |
| IUATLD management of tuberculosis: A Guide to Essential Practice ^17^ | Jaundice or severe abdominal discomfort | Not stated | Not stated |
| British HIV Association guidelines for the management of tuberculosis in adults living with HIV ^19^ | ALT > 3 x ULN with symptoms, or ALT > 5 X ULN without symptoms | Dose escalation, sequential | Yes |
| Clinical standards for the management of adverse effects during treatment for TB ^20^ | ALT > 3 x ULN with symptoms, or ALT > 5 X ULN without symptoms | Sequential | Yes |
| Up-to-Date: Treatment of drug-susceptible tuberculosis in non-pregnant adults without HIV infection ^21^ | AST > 3 X ULN with symptoms or > 5 X ULN without symptoms or bilirubin > 3 X ULN without symptom | Sequential | Not if severe |
| Management of drug-induced liver injury in people with HIV treated for tuberculosis: 2024 update (South Africa) ^22^ | ALT > 3 x ULN with symptoms or ALT > 5 X ULN without symptoms or ALT > 2 x baseline (in persons with abnormal baseline ALT) | Sequential | Only for patient with meningitis or intolerance to INH or RIF |
| MSF Medical Guidelines, Tuberculosis: Practical guide for clinicians, nurses, laboratory technicians and medical auxiliaries ^23^ | AST or ALT or bilirubin > 3 times ULN | Full re-challenge | No |
| Bangladesh ^24^ | ALT/AST > 5 times ULN (with or without jaundice), or jaundice (with or without increased ALT or AST levels) | Sequential | Not stated |
| Brazil ^25^ | ALT/AST > 5 times ULN (with or without jaundice), or jaundice (with or without increased ALT or AST levels) | Sequential | Not stated |
| China ^26^ | LFTS 5 x ULN or jaundice | Sequential | Not stated |
| Congo (Democratic Republic) ^47^ | Not stated | Sequential | Yes |
| Ethiopia ^27^ | Not stated | Sequential | No |
| Indonesia ^48^ | Not stated | Sequential | Not present |
| Kenya ^29^ | ALT or AST 5-10 x ULN | Sequential | No |
| Korea ^30^ | Not stated | Sequential | Yes |
| Liberia ^32^ | Jaundice or hepatitis (and other causes excluded) | Sequential | Not if jaundiced |
| Lesotho ^31^ | Jaundice or hepatitis (and other causes excluded) | Sequential | Not if jaundiced |
| Mozambique ^34^ | Not stated | Sequential | Not stated |
| Myanmar ^35^ | If liver disease thought to be caused by TB treatment | Sequential | Not if jaundiced |
| Namibia ^36^ | If liver enzymes > 3 x ULN and suspected drug-induced liver injury | Sequential | No |
| Nigeria ^37^ | Not stated | Not stated | Not stated |
| Pakistan ^38^ | Jaundice or hepatitis (and other causes excluded) | Not stated | Not stated |
| Philippines ^39^ | AST > 3 x ULN with symptoms, or > 5 x ULN without symptoms | Sequential | No |
| Sierra Leone ^40^ | Jaundice or signs of liver toxicity | Sequential | Not stated |
| South Africa ^41^ | Stop INH and RIF if symptoms or signs, or increased liver tests | Not stated | Not stated |
| Tanzania ^42^ | AST >3 x ULN with symptoms, or >5 x ULN without symptoms | Sequential | Not if severe |
| Thailand ^43^ | AST > 3 X ULN with symptoms, or > 5 X ULN without symptoms | Sequential | Not stated |
| Uganda ^44^ | Jaundice, AST > 3 x ULN | Sequential | Yes |
| Vietnam ^45^ | ALT > 3 x ULN with symptoms or ALT > 5 X ULN or ALT > 2 x baseline (in persons with abnormal baseline ALT) | Sequential | Not if severe |
| Zambia ^46^ | Jaundice severe or worsening, AST >3 x ULN with symptoms, or >5 x ULN without symptoms | Not stated | Not stated |

ATS/CDC/IDSA – American Thoracic Society, Centers for Disease Control and Prevention, Infectious Diseases Society of America

NICE – National Institute for Health and Care Excellence

IUATLD – International Union Against Tuberculosis and Lung Disease

MSF – Médecins Sans Frontières

**References for supplementary materials**

1. Keutzer L, Wicha SG, Simonsson US. Mobile Health Apps for Improvement of Tuberculosis Treatment: Descriptive Review. *JMIR Mhealth Uhealth* 2020; **8**(4): e17246.

2. Stop TB Partnership Childhood TB Subgroup, World Health Organization. Guidance for national tuberculosis programmes on the management of tuberculosis in children. Chapter 2: anti-tuberculosis treatment in children. *Int J Tuberc Lung Dis* 2006; **10**(11): 1205-11.

3. Tuberculosis Coalition for Technical Assistance. International Standards for Tuberculosis Care. 2006. <https://cdn.who.int/media/docs/default-source/documents/tuberculosis/istc_reportd3b3091a-acdb-487d-b2b0-83a3781ddcf6.pdf?sfvrsn=8f84bbe1_1&download=true> (accessed 19 November 2014).

4. Nahid P, Dorman SE, Alipanah N, et al. Official American Thoracic Society/Centers for Disease Control and Prevention/Infectious Diseases Society of America Clinical Practice Guidelines: Treatment of Drug-Susceptible Tuberculosis. *Clin Infect Dis* 2016; **63**(7): e147-e95.

5. Migliori GB, Sotgiu G, Rosales-Klintz S, et al. ERS/ECDC Statement: European Union standards for tuberculosis care, 2017 update. *Eur Respir J* 2018; **51**(5).

6. World Health Organization. WHO consolidated guidelines on tuberculosis: module 4: treatment: drug-susceptible tuberculosis treatment. 2022.

7. World Health Organization. WHO treatment guidelines for isoniazid-resistant tuberculosis: Supplement to the WHO treatment guidelines for

drug-resistant tuberculosis. 2018. <https://www.tbonline.info/media/uploads/documents/9789241550079-eng.pdf> (accessed 19 November 2024).

8. World Health Organization. WHO consolidated guidelines on tuberculosis: module 5: management of tuberculosis in children and adolescents. 2022. <https://www.who.int/publications/i/item/9789240046764>.

9. Johnston JC. Chapter 5: Treatment of tuberculosis disease. *Canadian Journal of Respiratory, Critical Care, and Sleep Medicine* 2022; **6**: 66–76.

10. Department of Health, Government of Australia. Tuberculosis: CDNA National Guidelines for Public Health Units. 2022. <https://www.health.gov.au/sites/default/files/documents/2022/06/tuberculosis-cdna-national-guidelines-for-public-health-units.pdf> (accessed 19 November 2024).

11. Panel de expertos de GeSIDA y de la División de Control de VIH I, Hepatitis , virales y Tuberculosis del Ministerio de Sanidad. Documento de consenso de GeSIDA/ División de Control de VIH, ITS, Hepatitis virales y Tuberculosis del Ministerio de Sanidad respecto al tratamiento antirretroviral en adultos infectados por el virus de la inmunodeficiencia humana. 2023. <https://gesida-seimc.org/wp-content/uploads/2023/06/Guia_TAR_V12.pdf> (accessed 19 November 2024 2024).

12. Panel on Guidelines for the Prevention and Treatment of Opportunistic Infections in Adults and Adolescents With HIV. Guidelines for the Prevention and Treatment of Opportunistic Infections in Adults and Adolescents With HIV. 12 November 2024 2024. <https://clinicalinfo.hiv.gov/sites/default/files/guidelines/documents/adult-adolescent-oi/guidelines-adult-adolescent-oi.pdf> (accessed 19 November 2024).

13. National Institute for Health and Care Excellence. Tuberculosis. 2024. <https://www.nice.org.uk/guidance/NG33> (accessed 19 November 2024).

14. Thwaites G, Fisher M, Hemingway C, et al. British Infection Society guidelines for the diagnosis and treatment of tuberculosis of the central nervous system in adults and children. *J Infect* 2009; **59**(3): 167-87.

15. British Thoracic Society Standards of Care Committee, Joint Tuberculosis Committee, Milburn H, et al. Guidelines for the prevention and management of Mycobacterium tuberculosis infection and disease in adult patients with chronic kidney disease. *Thorax* 2010; **65**(6): 557-70.

16. Gonzalez-Martin J, Garcia-Garcia JM, Anibarro L, et al. [Consensus document on the diagnosis, treatment and prevention of tuberculosis]. *Enferm Infecc Microbiol Clin* 2010; **28**(5): 297 e1-20.

17. Dlodlo RA BG, Heldal E, Allwood B, Chiang C-Y, Fujiwara PI, Graham SM, Guillerm N, Harries AD, Koura KG, Kumar AMV, Lin Y, Meghji J, Mortimer K, Piubello A, Roth B, Satyanarayana S, Sekadde M, Solovič I, Tonsing J, Van Deun A. Management of Tuberculosis: a Guide to Essential Practice. 2019. <https://theunion.org/sites/default/files/2020-08/TheUnion_Orange_2019.pdf> (accessed 19 November 2024).

18. European AIDS Clinical Society. Guidelines: version 11.1. 2022. <https://www.eacsociety.org/media/guidelines-11.1_final_09-10.pdf> (accessed 19 November 2024).

19. Bracchi M. British HIV Association guidelines for the management of tuberculosis in adults living with HIV 2018 (2023 interim update). 2023. <https://www.bhiva.org/file/5c485f3dc7c17/BHIVA-TB-guidelines.pdf> (accessed 19 November 2024).

20. Singh KP, Carvalho ACC, Centis R, et al. Clinical standards for the management of adverse effects during treatment for TB. *Int J Tuberc Lung Dis* 2023; **27**(7): 506-19.

21. Sterling TR. Treatment of drug-susceptible pulmonary tuberculosis in nonpregnant adults without HIV infection. 2024. <https://www.uptodate.com/contents/treatment-of-drug-susceptible-pulmonary-tuberculosis-in-nonpregnant-adults-without-hiv-infection#H2754992102> (accessed 19 November 2024 2024).

22. Boyles T, Berhanu RH, Gogela N, et al. Management of drug-induced liver injury in people with HIV treated for tuberculosis: 2024 update. *South Afr J HIV Med* 2024; **25**(1): 1558.

23. Varaine F, Hewison, C. MSF Medical Guidelines: Tuberculosis. 2024. <https://medicalguidelines.msf.org/en/viewport/TUB/english/tuberculosis-20321086.html> (accessed 19 November 2024).

24. Government of Bangladesh. National Guideline and Operational Manual for Tuberculosis: 6th Edition. 2021. <https://www.ntp.gov.bd/wp-content/uploads/2021/10/Operational-Manual-for-Tuberculosis_compressed.pdf> (accessed 11 December 2024).

25. Conde MB, Melo FA, Marques AM, et al. III Brazilian Thoracic Association Guidelines on tuberculosis. *J Bras Pneumol* 2009; **35**(10): 1018-48.

26. Office of the National Health Commission of the People's Republic of China. China Tuberculosis Prevention and Control Technical Specifications. 2020.

27. Ministry of Health, Government of Ethiopia. Guidelines for Management of TB, DR-TB and Leprosy in Ethiopia. 6th edition 2017. <https://www.afro.who.int/publications/national-guidelines-tb-drug-resistant-tb-and-leprosy-ethiopia-sixth-edition> (accessed 11 December 2024).

28. Ministry of Health and Family Welfare, India. INDEX-TB Guidelines. Guidelines on extra-pulmonary tuberculosis for India. . 2016. <https://vikaspedia.in/health/diseases/infectious-diseases/tuberculosis/standards-for-tb-care-in-india> (accessed 11 December 2024).

29. Ministry of Health, Government of Kenya. Integrated guideline for tuberculosis, leprosy, and lung disease. 2021. <https://chskenya.org/wp-content/uploads/2022/04/INTEGRATED-GUIDELINE-FOR-TUBERCULOSIS-LEPROSY-AND-LUNG-DISEASE-2021.pdf> (accessed 11 December 2024).

30. Joint Committee for the Revision of the Korean Guidelines for Tuberculosis in collaboration with the Korea Centers for Disease Control and Prevention. Korean Guidelines For Tuberculosis. 2024.

31. Ministry of Health, Government of Lesotho. National Guidelines for Drug Susceptible Tuberculosis: 2019 Edition. 2019. <http://health.gov.ls/download/tb-guidelines-2019/> (accessed 11 December 2024).

32. Ministry of Health, Liberia. National Tuberculosis Management Guidelines, Liberia. 2019.

33. Ministry of Health, Mongolia. Mongolia TB treatment guidelines. 2009.

34. Ministry of Health, Mozambique. Mozambique National TB Protocols. 2019.

35. Ministry of Health and Sports, Government of Myanmar. Guideline for Drug Sensitive TB Management in Myanmar. 2023. <https://aislearningplatform.org/resource/guideline-for-drug-sensitive-tb-management-in-myanmar/> (accessed 11 December 2024).

36. Ministry of Health, Government of Namibia. National Guidelines for the Management of Tuberculosis Fourth Edition 2019. <https://policyvault.africa/policy/national-guidelines-for-the-management-of-tuberculosis/> (accessed 11 December 2024).

37. Department of Public Health National Tuberculosis and Leprosy Control Programme, Nigeria. National Tuberculosis, Leprosy and Buruli Ulcer Management and Control Guidelines. 2015. <https://ntblcp.org.ng/technical-guidelines/> (accessed 11 December 2024).

38. Government of Pakistan. National guidelines for the control of tuberculosis in Pakistan. 2019. <http://ntp.gov.pk/ntp-old/uploads/National_Guidelines_for_TB_Revised_2019.pdf> (accessed 11 December 2024).

39. Department of Health - National Tuberculosis Control Program, the Phillipines. TB (DS & DR) and Latent TB Screening, Diagnosis and Management Pocket Guide. 2023. <https://ntp.doh.gov.ph/resources/downloads/publications/guidelines/> (accessed 11 December 2024).

40. Ministry of Health, National Leprosy and Tuberculosis Control Programme. National Guidelines for Clinical and Programmatic Management of TB in Sierra Leone. 2024.

41. Department of Health, Republic of South Africa. National guidelines on the treatment of tuberculosis infection. 2023. <https://knowledgehub.health.gov.za/system/files/elibdownloads/2023-04/Health_Latent%2520TB%2520Infection_2023_web.pdf> (accessed 11 December 2024).

42. Ministry of Health, Government of Tanzania. Manual for management of tuberculosis and leprosy in Tanzania. 2020. Manuals and Guidelines | National Tuberculosis & Leprosy Programme (ntlp.go.tz) (accessed 11 December 2024).

43. Ministry of Health, Government of Thailand. Thailand National Tuberculosis Control Program Guideline. 2018. <https://www.pidst.or.th/A641.html?action=download&file=750_Nation%20TB%20guideline%202018.pdf> (accessed 11 December 2024).

44. Ministry of Health, Uganda. Uganda National Tuberculosis and Leprosy Control Programme Manual for Management and Control of Tuberculosis and Leprosy. 3rd Edition. 2017. NTLP-Manual-3rd-edition_17th-Aug_final.pdf (health.go.ug) (accessed 11 December 2024).

45. Ministry of Health, Vietnam. Guidelines for Diagnosis, Treatment, and Prevention of Tuberculosis. 2024.

46. Ministry of Health, Republic of Zambia. National tuberculosis and leprosy programme. 2022. <https://www.moh.gov.zm/wp-content/uploads/filebase/guidelines/tb_guidelines/4.-Consolidated-TB-Guidelines-for-Zambia-Final-Version.pdf> (accessed 11 December 2024).

47. Ministère de la Santé Publique, Hygiène et Prévention in the Democratic Republic of Congo. Guide de Prise en Charge de la Tuberculose. 2022. <https://www.tbdiah.org/wp-content/uploads/2023/11/PATI-6_-VERSION-DU-26_02_2022-KISANTU-1-1-1.pdf> (accessed 11 December 2024).

48. Menteri Kesehatan, Republik Indonesia. Indonesian Health Ministrial Decree No.67 year 2016 (National TB Guidelines). 2016. <http://impaact4tb.org/download/indonesia-health-ministerial-decree-2016-national-tb-guidelines/?wpdmdl=6139&refresh=6759e6af0d64b1733945007> (accessed 11 December 2024).

49. Blumberg HM, Burman WJ, Chaisson RE, et al. American Thoracic Society/Centers for Disease Control and Prevention/Infectious Diseases Society of America: treatment of tuberculosis. *Am J Respir Crit Care Med* 2003; **167**(4): 603-62.

50. Migliori GB, Zellweger JP, Abubakar I, et al. European union standards for tuberculosis care. *Eur Respir J* 2012; **39**(4): 807-19.

51. World Health Organization. Treatment of tuberculosis: Guidelines, Fourth edition. 2010. <https://iris.who.int/bitstream/handle/10665/44165/9789241547833_eng.pdf?sequence=12024>).

52. World Health Organization. WHO consolidated guidelines on tuberculosis. Module 4: treatment - drug-susceptible tuberculosis treatment. 2022. <https://www.who.int/publications/i/item/9789240048126> (accessed 19 November 2024).
